# Imported *Plasmodium falciparum *malaria in HIV-infected patients: a report of two cases

**DOI:** 10.1186/1475-2875-11-136

**Published:** 2012-04-27

**Authors:** Silvia García-Bujalance, Carolina Navarro-San Francisco, José M Rubio, José R Arribas, Avelino Gutierrez

**Affiliations:** 1 Department of Microbiology and Parasitology, La Paz University Hospital-IdiPAZ, Paseo de la Castellana 261, 28046 Madrid, Spain; 2 Infectious Diseases and Clinical Microbiology Unit, Division of Internal Medicine and Division of Microbiology, La Paz University Hospital-IdiPAZ, Paseo de la Castellana 261, 28046 Madrid, Spain; 3 Malaria and Emerging Parasitic Diseases Laboratory, Parasitology Department, Nacional Centre of Microbilogy, Instituto de Salud Carlos III, Cra. Majadahonda Pozuelo Km 2, Majadahonda, 28220 Madrid, Spain; 4 HIV Unit and Infectious Diseases and Clinical Microbiology Unit, Division of Internal Medicine and Division of Microbiology, La Paz University Hospital-IdiPAZ, Paseo de la Castellana 261, 28046 Madrid, Spain

**Keywords:** *Plasmodium falciparum*, HIV, Imported malaria, Sequence analysis, Drug interaction

## Abstract

As HIV becomes a chronic infection, an increasing number of HIV-infected patients are travelling to malaria-endemic areas. Association of malaria with HIV/AIDS can be clinically severe. Severe falciparum malaria is a medical emergency that is associated with a high mortality, even when treated in an Intensive Care Unit. This article describes two cases of HIV-positive patients, who returned from malaria-endemic areas and presented a parasitaemia > 5% of erythrocytes and clinical signs of severe falciparum malaria, both with > 350 CD4 cell count/μl, absence of chemoprophylaxis and successful response. Factors like drug interactions and the possible implication of anti-malarial therapy bioavailability are all especially interesting in HIV-malaria co-infections.

## Background

*Plasmodium falciparum *imported malaria causes severe clinical episodes every year in European travellers. Between 2% and 16% of these infections are severe cases according to the World Health Organization (WHO) definition [1-3]. Currently, the travellers' profile presents great diversity. An increasing number of patients with human immunodeficiency virus (HIV) are travelling to malaria-endemic areas [4]. Studies about countries where malaria is endemic suggest that the patients with a low CD4 T cell count or advanced HIV-1 disease had an increased risk of malaria events, higher parasitaemia and more severe clinical episodes [5-9]. Other authors have reported that the impact of HIV infection on the severity of an imported malaria infection is restricted to patients with CD4 cell counts < 350 cells/μL [10]. Therefore, factors such as the interaction between anti-malarial drug and anti-retroviral therapy are important in the management of clinical malaria episodes. Artemisinin-based combination therapies and a parenteral therapy combination with artesunate are now accepted as the first-line recommendations by WHO for the treatment of uncomplicated and severe malaria, respectively. However, there are no anti-malarial treatment guidelines for HIV-infected patients. Parasitological response to treatment of acute malaria among HIV-sero-positive individuals has not been evaluated [11].

Two clinical cases of imported *P. falciparum *malaria in two HIV-infected patients are presented and discussed, because of the possibility of improved diagnosis methods needed on admission or for follow-up anti-malarial therapy.

## Case presentation

### Case report 1

A 47-year-old man from Spain presented in University Hospital La Paz in Madrid, Spain, with a two-day history of intermittent fever, headache and fatigue. The patient had returned a week earlier from a four-week trip to Equatorial Guinea. He did not take anti-malarial chemoprophylaxis during the visit. He was a category A3 HIV-infected patient with a CD4 cell count of 650/μL and HIV viral load of less than 20 copies/mL. He had been taking tenofovir, emtricitabine and efavirenz since 2008. On examination, he was febrile (38.7°C) and had a heart rate of 120 beats/min, blood pressure of 93/64 mmHg, normal respiration rate and oxygen saturation 95% on room air. Laboratory investigations showed normal haemoglobin concentration (14.8 gr/dL), normal cell count and leukocyte formula (4.4 × 10^9 ^cells/L, N 89.1%, L 6.9% M 1.6%), moderate thrombocytopaenia (36 × 10^9 ^cells/L, reference range 125-350 × 10^9 ^cells/L), normal glucose levels (110 mg/dL), bilirubin (28 μmol/L, reference value < 20 μmol/L), creatinine (176 μmol/L, reference range 40-120 μmol/L) and slightly increased aspartate transaminase (82 UI/L, normal < 37 UI/L) and an elevated C-reactive protein concentration of 157 mg/L (normal < 10 mg/L). Malaria parasites were seen on Giemsa-stained thick and thin blood films with Field's stain in 7% of erythrocytes. Parasite morphology identified *P. falciparum*. A rapid diagnostic test (RDT) result for histidine-rich protein 2 of *P. falciparum *(Now^® ^Malaria Test, Binax INC, Maine, USA) was positive. Results of multiplex PCR assay, species-specific nested-PCR [12] were positive for *P. falciparum*. The patient was admitted to the intensive care unit with several signs of severe malaria (parasitaemia of 7% of erythrocytes, haemodynamic instability, decreased level of consciousness, respiratory distress syndrome and mild renal insufficiency) for monitoring. Patient was treated with quinine and doxycycline intravenous for seven days at the recommended doses in adults. The anti-retroviral therapy with tenofovir, emtricitabine and efavirenz was not interrupted. On day 2 the parasitaemia was 1%. The parasites were cleared after six days without recrudescence. The hospital stay lasted 13 days.

### Case report 2

A 38-year-old man from France presented to University Hospital La Paz, with a two-day history of fever, sweating, abdominal pain and vomiting after returning from a six-day visit to the Ivory Coast. He did not use personal vector avoidance measures (insect repellent, long-sleeved clothing, netting). He had not taken anti-malarial chemoprophylaxis. He had been diagnosed with HIV infection 10 years before. He was taking tenofovir, abacavir and darunavir/ritonavir with a CD4 cell count of 432/μL and undetectable viral load (less than 20 copies/mL). He was admitted to the emergency department with a temperature of 39.9°C but he was in good general condition with a normal respiratory rate, blood pressure of 147/88 mmHg, and normal oxygen saturation. The day of admission, laboratory investigations revealed: normal haemoglobin concentration (15,0 gr/dL), normal white cell count and leukocyte formula (6.3 × 10^9 ^cells/L, N 78.3%, L 12.3%, M 8.5%), moderate thrombocytopaenia (91 × 10^9 ^cells/L, reference range 125-350 × 10^9 ^cells/L), normal glucose levels (104 mg/dL), bilirubin (1.6 μmol/L, reference value < 20 μmol/L), creatinine (135 μmol/L, reference range 40-120 μmol/L) and slightly increased aspartate transaminase (95 UI/L, normal < 37 UI/L) and ALT (92 UI/L, normal < 65 UI/L) and an elevated C-reactive protein concentration of 112 mg/L (normal < 10 mg/L). Malaria parasites were detected by microscopic examination in 0.5% of his erythrocytes. The RDT (Now^® ^Malaria Test, Binax INC, Maine, USA) was positive for *P. falciparum*. The patient was treated with atovaquone/proguanil 250 mg/100 mg, every six hours for three days. On day 3, he was again febrile with *P. falciparum *parasitaemia nearly 8% of erytrocytes. His laboratory results showed a decreased haemoglobin concentration of 10.0 gr/dL and thrombocytopaenia more severe than 33 × 10^9 ^cells/L. Anti-malarial treatment was changed to intravenous quinine and doxycycline for seven days and was monitored in the intensive care unit for the first 24 hours. The anti-retroviral therapy with tenofovir, abacavir and darunavir/ritonavir was stopped, after initiating the malarial treatment with quinine. On day 6 he was cleared of parasitaemia. He was discharged after 10 days. The multiplex PCR assays performed on the sample collected on the day of admission confirmed that the patient was only infected with *P. falciparum*. Analysis of point mutations in the cytochrome b gene, related to resistance to atovaquone, and in the dihydrofolate reductase (DHFR) gene, related to proguanil resistance, show a single mutation in the cytochrome b gene (L283I) and four point mutations for the DHFR gene (Table 1).

**Table 1:** Molecular marker codons

	*Dhfr *genotype					*Pfcyt b *genotype						
aa position	16	**51**	**59**	**108**	**165**	133	268	272	275	280	**283**	284

Wild type	A	N	C	S	G	M	V	K	P	G	L	V

Mutant type	V	I	R	N/T	K	I	S/C	R	T	D	I	K

Case report(2)	A	**I**	**R**	**N**	**K**	M	V	K	P	G	**I**	V

## Conclusions

*Plasmodium falciparum *parasitaemia is higher in HIV-infected patients, especially in those with a low CD4 cell count [5-10]. In the first case, the patient had a parasitaemia of 7% in the blood smear at the time of diagnosis and a 650 CD4 T cell count/μl. A parasitaemia above 5% in non-immune travellers for malaria falls within the severity criteria according to WHO definition. Older age, European origin, travel to eastern Africa, absence of chemoprophylaxis, time to diagnosis of four to 12 days and diagnosis during the fall-winter season have been associated with severe imported *P. falciparum *malaria in non-immune travellers [2]. Data about the impact of HIV-1 infection on the severity of imported *P. falciparum *malaria have been reported and the most frequent criterion of severity was hyperparasitaemia [10]. There are few clinical trials on the co-administration of anti-retroviral therapy and anti-malarial drugs [13]. Interactions between quinine and nevirapine or ritonavir, including ritonavir-boosted protease inhibitor regimens have been reported. The concurrent administration of nevirapine and quinine can lead to significant reductions in the efficacy of the quinine and increased toxicity [14]. The co-administration of ritonavir and quinine can lead to elevations in plasma levels of quinine and suggest that quinine dosages should be shifted downward [15]. Anti-retroviral drugs are the most therapeutically risky drugs for drug-drug interactions because of their potent inhibition and induction of cytochrome enzymes. This is particularly important during co-administration of lumefantrine (in combination with artemisinin derivates) and protease inhibitors or non-nucleoside reverse transcriptase inhibitor-based anti-retroviral regimens [16]. The first patient received intravenous quinine and doxycycline with a successful therapeutic response. Quinine therapy was used because in Europe the available formulations of intravenous artesunate recommended as the first-line by WHO were not produced according to Good Manufacturing Practices (GMP). WHO recently pre-qualified intravenous artesunate manufactured by Guilin Pharmaceuticals in China and this may resolve problems in acquiring GMP artesunate in Spain [17].

In the second case, the patient was initially treated with atovaquone/proguanil. Atovaquone-proguanil has shown high efficacy against multi- drug-resistant *P. falciparum *with few side effects and a mode of action unrelated to that of other anti-malarial drugs [18], such as quinine, plus one of the three following: doxycycline, tetracycline or clindamycin and mefloquine. The patient developed an early treatment failure (within 72 hours) with an elevation of parasitaemia and a jump from 0.5% to 8% infected erythrocytes in the thick blood film. The malarial treatment failures can be attributed to recrudescence of *P. falciparum *due to suboptimal therapy or anti-malarial drug-resistant genotypes. Atovaquone is a low genetic barrier drug but proguanil synergizes with atovaquone in combination, reducing the selection of resistance mutations. Resistance to atovaquone and proguanil has been associated with sequence changes in cytochrome b (*cytb*) and dihydrofolate reductase (*dhfr*) gene respectively. Isolates of *P. falciparum *collected by the European Network on Imported Infectious Disease surveillance were analysed for single-point mutations of the *cytb *gene and the results showed a level < 1% of these mutations [19]. Atovaquone inhibits electron transfer of the respiratory chain by binding to cytochrome b of plasmodial mitochondria. On the other side, proguanil, as its active form cycloguanil, is an inhibitor of the DHFR involved in pyrimidine biosysnthesis. However, in combination with atovaquone, by an unknown mechanism, it lowers the effective concentration at which the former collapses the mitochondrial membrane potential [20]. In the second case, the analysis of mutations of the DHFR and cytochrome b genes showed the characteristic triple DHFR mutations associated with failure for the treatment of pyrimethamine, but not of proguanil. The *cytb *gene showed the mutation L283I. This mutation, associated with V284K, shows a 76-fold increase in IC_50 _of atovaquone compared to non-mutant strains, but this effect must be the direct responsibility of V284K, as I283 has been observed in atovaquone sensitive parasites [21]. The presence of the L283I mutation by itself does not confer resistance to atovaquone. These data suggest that the early treatment failure in the second patient, could be more related to a suboptimal therapy than to a drug-resistant genotype. Atovaquone/proguanil is used in the treatment of uncomplicated multi- drug-resistant *P. falciparum *malaria, but we do not have an answer to this question: could the HIV-infected patients have also a higher rate of atovaquone/proguanil treatment failure? No data about this point have been reported in HIV-infected patients. This question must be approached with caution. An explanation could be a delayed parasite clearance due to a slowness of action by atovaquone/proguanil, but the parasitaemia increased from 0.5% to 8% on day 3. On the other hand, treatment failure could be attributed to poor bioavailability of the atovaquone, proguanil or its active metabolite, cycloguanil. The biotransformation of proguanil could have been reduced. A possible interaction between atovaquone/proguanil and IP-based regimens could be presented [22]. However, it was not possible to measure drug concentrations at that time.

In routine clinical practice, the PCR is performed on admission samples to confirm the results obtained by microscopy, as shown in this cases, and to avoid the limitations of the microscopy and RDT: misdiagnosis of mixed-species infections and low parasitaemia. The microscopic examination of Giemsa-stained thin and thick blood films remains the "gold standard" for the diagnosis of malaria and detects parasites at a density of 50 parasites/μl by an experienced microscopist [23]. The RDT, using a protein rich in histidine (HRP-2) and aldolase or lactate dehydrogenase antigens, detects parasites quickly and cheaply, but it cannot provide data regarding drug resistance genotypes. RDT is accepted as an excellent tool in the diagnosis of *P. falciparum *malaria. However, it is not useful in the follow-up anti-malarial therapy, because it could positive HRP-2 antigen for three or four weeks after a successful therapy. The PCR-based methods are extremely sensitive with a detection limit of < 10 parasites/μl. Various authors have reported the development of a real-time PCR assay to detect *P. falciparum, Plasmodium vivax *and *Plasmodium ovale *in routine clinical diagnosis [24]. The risks of contamination are minimal and the result can be obtained in only 2 h. In the cases presented, molecular methods were essential for rapid and accurate diagnosis and follow-up.

In conclusion, this are two cases of HIV-infected patients with imported *P. falciparum *malaria, high parasitaemia, both severe clinical episodes with > 350 CD4 cell count/μl, absence of chemoprophylaxis and successful response. Factors such as drug interactions and the possible implication of anti-malarial therapy bioavailability are all especially interesting in HIV-malaria co-infections.

## Consent

Written informed consent was obtained from the patient for publication of this Case report. A copy of the written consent is available for review by the Editor-in-Chief of this journal.

## Competing interests

The authors declare that they have no competing interests.

## Authors' contributions

SGB, CNSF, JMR, JRAL and AGA conceived this case report and wrote the paper. CNSF and JRAL participated in the medical care and follow-up of the patients. SGB, CNSF, drafted the manuscript and collect data. JMR was responsible for molecular detection and analysis of sequence data. All authors have read and approved the final manuscript.
